# Acute Immune-Inflammatory Responses to a Single Bout of Aerobic Exercise in Smokers; The Effect of Smoking History and Status

**DOI:** 10.3389/fimmu.2015.00634

**Published:** 2015-12-23

**Authors:** Tegan Emma Kastelein, Rob Duffield, Frank E. Marino

**Affiliations:** ^1^School of Human Movement Studies, Charles Sturt University, Bathurst, NSW, Australia; ^2^Sport and Exercise Discipline Group, Faculty of Health, University of Technology Sydney, Sydney, NSW, Australia

**Keywords:** cycling, cytokines, leukocytes, smokers, non-smokers

## Abstract

This study examined the acute immune and inflammatory responses to exercise in smokers compared to non-smokers, and further, the effect of smoking history on these immune-inflammatory responses. Fifty-four recreationally active males who were either smokers (SM; *n* = 27) or non-smokers (NS; *n* = 27) were allocated into either young (YSM, YNS) or middle-aged groups (MSM, MNS) based on smoking status. Participants were matched for fitness and smoking habits and following familiarization and baseline testing, undertook an exercise protocol that involved 40 min of cycle ergometry at 50% of VO_2peak_. Venous blood was obtained pre- and post- (0 min, 1, and 4 h) exercise to measure circulating leukocytes and inflammatory markers interleukin (IL)-6, IL-1β, IL-1ra, and monocyte chemoattractant protein-1 (MCP-1). Compared to MNS, MSM showed elevated basal concentrations of MCP-1, which were increased with a longer smoking history (*P* < 0.05). In response to exercise, YSM demonstrated an amplified IL-6 response from immediately- to 1 h-post compared to YNS. Furthermore, IL-1ra in YSM was elevated above that of YNS across all time points (*P* < 0.05). The MSM group had higher IL-1β at baseline when compared to YSM, although IL-1ra was greater for YSM at baseline (*P* < 0.05). Finally, the post-exercise leukocyte response was greater in MSM compared to YSM and non-smokers (*P* < 0.05). In conclusion, smoker’s exhibit elevated MCP-1 and IL-1β that seem to be evident with a longer smoking history (~15 years). Furthermore, the differences in exercise-induced inflammatory responses noted in YSM may be indicative tobacco smoke exposure priming circulating leukocytes to amplify inflammatory responses.

## Introduction

Long-term tobacco smoking induces a myriad of inflammatory processes and alterations to the immune system, which in turn are key steps in the pathogenesis of cardiovascular disease (CVD), diabetes, and chronic obstructive pulmonary disease (COPD) (1–3). Specifically, long-term smoking is reported to up-regulate pro-inflammatory cytokines, including interleukin (IL)-6, IL-1 beta (IL-1β), tumor necrosis factor-alpha (TNF-α), and C-reactive protein (CRP). In turn, this pro-inflammatory up-regulation results in the development of a low grade systemic inflammatory state, which presents as an independent risk factor for disease development (4–6). While immune and inflammatory changes are suggested to result from a lifetime of smoking (4, 5), the acute physiological outcomes of a shorter smoking history are unknown. Conversely, exercise is viewed as an effective regulator of immune function (7), particularly given its ability to promote an anti-inflammatory environment (8–10). Consequently, the exercise-induced anti-inflammatory response may be beneficial for long-term smokers, although research outlining this remains scarce. Given that immune and inflammatory changes associated with smoking are suggested to precede chronic disease development (2, 11), and exercise has been reported as an effective tool in meditating such developments (12, 13), it is pertinent to determine inflammatory responses to exercise in a smoker population.

While smoking induces both immunosuppressive and inflammatory effects resulting in the development of the aforementioned pathological states (1, 14), regular exercise is reported as an effective intervention for mediating disease risk (12, 13). While some exercise-induced physiological responses of smokers have been reported, including heart rate (HR), cardiovascular function, and oxygen consumption (15–17), the immune-inflammatory parameters remain less well defined. In healthy non-smokers, an acute bout of exercise has been shown to result in augmented inflammatory cytokines, including IL-6, IL-1ra, and IL-10 with anti-inflammatory actions (12); the magnitude of which is dependent on modality, intensity, and duration (9). Thus, given the elevated pro-inflammatory state of smokers [IL-1β, monocyte chemoattractant protein-1 (MCP-1), and IL-6], coupled with the proposed anti-inflammatory response to moderate-intensity exercise (Il-1ra and IL-6), it remains unknown of the exercise response is blunted in smoking populations, or with prolonged smoking exposure.

Despite the well-known effects of exercise on immune and inflammatory regulation and the deleterious effect of long-term smoking, few have sort to investigate the effects of exercise in smokers who may exhibit altered immune responses. Flouris et al. (18) suggest that even brief exposure to second hand smoke (SHS) markedly reduced cardiorespiratory and immune variables following 30 min of moderate intensity exercise (18). Given that long-term smoking is associated with immunosuppression, increased inflammation, and thus greater susceptibility to disease development, it seems particularly important to examine the effects of the exercise-induced inflammatory response in smokers. Furthermore, given the lack of longitudinal cohort data, the observed effect in those with relatively shorter compared to longer smoking histories also seems pertinent. That is, it is unknown whether smokers with a shorter smoking history present the immune alterations as observed in long-term smokers, while still matching for factors that can affect inflammatory state, such as aerobic fitness. Therefore, this study aims to determine the acute immune and inflammatory responses to exercise in smokers compared to non-smokers. A secondary aim is to examine the effect of smoking history on the immune-inflammatory responses.

## Materials and Methods

### Participants

The study population consisted of 54 recreationally active males who were either smokers (SM; *n* = 27) or non-smokers (NS; *n* = 27). Participants were allocated into either young (YSM, YNS) or middle-aged groups (MSM, MNS) based on the duration of their smoking status. In the current study, delimitations for age were presented, while a number of middle-aged smokers were recruited, ~10 were excluded as they presented with pre-existing medical conditions and were not appropriate to participate in the study. Accordingly, participants reported as apparently healthy and free from any known metabolic, cardiovascular, or pulmonary disease, immunological irregularities or other conditions associated with systemic inflammatory responses and no participants were taking any potentially confounding medications. Participants were matched for age and aerobic fitness, with anthropometric and descriptive baseline values reported in Table 1. The NS participants (YSM and MNS) were classified as never smokers and were otherwise healthy individuals who reported modest regular physical activity (2–3 days of 30 min/week). The smokers (YSM and MSM) were otherwise healthy individuals who were current active smokers. The self-reported smoking history for the SM population was 5.2 ± 1.7 years of smoking and 12.3 ± 6.5 cigarettes/day for YSM and 14.6 ± 6.3 years of smoking and 15.8 ± 7.3 cigarettes/day MSM. Smoking participants engaged in comparable levels of recreational physical activity as the non-smokers based on qualitative feedback regarding exercise engagement. Prior to the commencement of the study, all participants were required to provide written and verbal consent following an outline of all procedures and measures. This study conformed to the Declaration of Helsinki and was approved by the Research in Human Ethics Committee at the University.

**Table 1:** **Mean baseline anthropometric, biochemistry, aerobic fitness, and smoking variables within the smoking (*n* = 27; YSM = 14, MSM = 13) and non-smoking (*n* = 27; YNS = 14, MNS = 13) populations**.

Anthropometric data	Young smokers	Young non-smokers	Middle-aged smokers	Middle-aged non-smokers
Age (years)	22.00 ± 1.57	22.00 ± 1.60	33.00 ± 7.75*	36.00 ± 6.59^#^
Height (cm)	182.00 ± 0.07	182.00 ± 0.06	177.00 ± 0.07	178.00 ± 0.06^#^
Weight (kg)	81.78 ± 12.07	86.88 ± 16.5	81.22 ± 12.87	90.83 ± 14.51
VO_2_ peak (mL. kg^−1^. min^−1^)	36.67 ± 2.94	39.27 ± 6.06	33.93 ± 8.42	31.62 ± 5.96
Waist to hip ratio	0.86 ± 0.05	0.85 ± 0.10	0.86 ± 0.06	0.91 ± 0.07^#^
SBP (mmHg)	113.64 ± 9.79	106.71 ± 28.54	116.27 ± 10.08	116.75 ± 9.28
DBP (mmHg)	69.46 ± 9.10	69.29 ± 9.54	76.80 ± 8.91	77.77 ± 8.63
Heart rate (bpm)	69.14 ± 11.02	75.33 ± 11.26	65.43 ± 12.45	66.62 ± 8.26
% Fat mass	15.62 ± 5.78	17.5 ± 8.14	24.75 ± 6.76*	26.51 ± 4.96^#^
Lean mass (kg)	63.0 ± 9.05	65.55 ± 12.12	59.02 ± 6.61	61.23 ± 5.90
Fat mass (kg)	12.37 ± 5.32	18.22 ± 10.15	20.68 ± 6.83*	24.96 ± 7.01
**Biochemistry**				
CRP (mg/L)	2.00 ± 1.89	0.92 ± 0.52*	1.98 ± 1.79	1.30 ± 0.92
HDL (mmol L^−1^)	1.06 ± 0.034	1.19 ± 0.27	1.22 ± 0.36	1.12 ± 0.25
Triglycerides (mmol L^−1^)	1.28 ± 0.64	0.99 ± 0.33	1.50 ± 0.84	1.28 ± 0.37^#^
Fasting glucose (mmol L^−1^)	4.21 ± 0.82	5.18 ± 0.55	5.36 ± 0.40	4.53 ± 0.90
**Smoking variables**				
Smoking history (years)	5.21 ± 1.71		14.62 ± 6.29	
Pack-years	2.86 ± 1.72		12.15 ± 9.08	
Cigarettes/day	12.31 ± 6.54		15.79 ± 7.33	
Fagerstrom dependence score	2.31 ± 1.32		2.64 ± 1.28	

*Denotes statistically different to YSM (P < 0.05).

*^#^Denotes statistically different to YNS (P < 0.05)*.

### Study Overview

Prior to involvement, participants were required to undergo a medical screening, complete an adult pre-exercise screening system (APSS), a healthy history questionnaire and the Fagerstrom test for nicotine dependence [FTND; (19)]. If participants satisfied the inclusion criteria, they were then enrolled in the study. A familiarization of all measures and procedures and then baseline testing sessions were conducted to obtain anthropometric and aerobic exercise capacity data (VO_2peak_). Following ~7 days’ rest, participants returned to the laboratory for a testing session. Participants were required to cycle at 50% of VO_2peak_ for the duration of 40 min. HR and rating of perceived exertion (RPE; Borg CR10 scale; Borg 1990) were collected throughout the 40 min. Additionally, a venous blood sample was obtained pre- and post- (0 min, 1, and 4 h) exercise to measure circulating leukocytes and inflammatory markers (IL-6, IL-1β, IL-1ra, and MCP-1). For the 48-h prior to all testing procedures, participants were required to abstain from strenuous physical activity. Furthermore, 10 h prior to testing participants underwent an overnight fast, which also included abstinence from cigarette smoking, caffeine, and alcohol (as confirmed from self-reported diaries).

### Baseline Testing

Participants reported to the laboratory between 0500 and 0800 h, rested and fasted for a baseline testing session. Stature (Stadiometer: Custom CSU, Bathurst, NSW, Australia), body mass (HW 150 K, A and D, Bradford, MA, USA), and waist and hip circumferences (steel tape, EC P3 metric graduation, Australia) were obtained for analysis of body composition based on standardized techniques (20). Body mass index (BMI) was calculated from mass and stature, further waist and hip circumferences provided a waist to hip ratio. In addition, a supine dual-energy x-ray absorptiometry (DXA) scan was conducted for the determination of body composition (XR800, Norland, Cooper Surgical Company, Trumbull, CT, USA). Scanning resolution and speed were set at 6.5 mm × 13.0 mm and 130 mm s^−1^, respectively. Whole body scans were analyzed (Illuminatus DXA, ver. 4.2.0, USA) for total body lean mass and total body fat mass and are reported in absolute and relative terms. Resting blood pressure was obtained through a commonly used indirect technique involving the use of an aneroid sphygmomanometer and stethoscope (Welch-Allyn, Arden, NC, USA); furthermore, participants were fitted with a HR monitor (Rs800cx, Vantage NV, Polar, Finland) to obtain a measure of resting HR. Additionally, a baseline blood sample was collected to determine fasting glucose, total cholesterol, and baseline immune and inflammatory markers.

Participants also performed a graded exercise test (GXT) on an electronically braked cycle ergometer (LODE Excalibur Sport, LODE BV, Groningen, The Netherlands) for the determination of VO_2peak_. In order to accommodate different likely physical capacities, the younger population began the incremental GXT at 100 W and increased by 25 W every minute until volitional exhaustion, whereas the middle-aged population began the GXT at 25 W and increase 25 W every minute. HR was obtained every minute and a session RPE (Modified Borg CR10 scale) was collected at completion of the GXT. Pulmonary gas exchange was measured by determining O_2_ and CO_2_ concentrations and ventilation to calculate VO_2_ using a metabolic gas analysis system (Parvo-Medics, True2400, East Sandy, UT, USA). The system was calibrated according to the manufacturer’s instructions. This involved the pneumotachometer calibration using a 3-L syringe. The gas analyzers were calibrated using a two-point fully automated process involving room air and gas calibration for fractional gas concentration with a gravimetric gas mixture of known concentrations [CO_2_, 4.1% (0.1); O_2_, 15.7% (0.2)].

### Experimental Protocol: Acute Exercise

Following the baseline session, participants reported to the laboratory in a fasted and rested state for the completion of the exercise protocol that consisted of 40 min of stationary cycle ergometry (Monark 828E, Monark Exercise AB, Varburg, Sweden) at 50% of VO_2peak_. The workload was calculated as 50% of the pedaling resistance (Watt) achieved during the GXT and was converted into kilopond units and set as a fixed intensity for the exercise protocol. The selection of this exercise protocol was based on previous research (21) that demonstrated an inflammatory response to an acute bout of exercise of the same intensity and duration as the current study, yet within the tolerable limits for middle-aged participants of lower fitness capacities. Telemetry-based HR (Rs800cx, Vantage NV, Polar, Finland) and RPE were recorded every 5 min during the exercise protocol.

### Blood Collection

Venous blood was collected pre- and post- (0 min, 1, and 4 h) exercise via a 20-guage catheter inserted into the medial antecubital vein. Approximately 40 mL of blood was collected and aliquoted into serum separator tubes (SST) for analysis of baseline blood lipid profile and CRP, ethylene diamine tetraacetic acid (EDTA) tubes for analysis of IL-6, IL-1ra, IL-1β, MCP-1, glycosylated hemoglobin (HbA1c), glucose, lactate, and total and sub-population leukocyte count. EDTA tubes were centrifuged immediately post-aliquot at 3500 rpm for 15 min at 4°C, and SST tubes were left to clot at room temperature for 20 min prior to centrifugation. Supernatants were immediately stored at −80 and −20°C for EDTA and SST, respectively.

### Blood Analysis

Blood samples were analyzed for blood lipid profile, CRP, IL-6, IL-1ra, IL-1β, MCP-1, and total and sub-population leukocyte count. Total cholesterol was analyzed using an enzymatic method and polychromatic endpoint technique measurement (Dimension Xpand Plus, Siemens Healthcare Diagnostics, Sydney, NSW, Australia). HDL cholesterol was measured using accelerator selective detergent methodology. Triglycerides were assessed using an enzymatic method and biochromatic endpoint technique measurement. Furthermore, HbA1c was measured using automated high-performance liquid chromatography methodology (Bio-Rad Variant, Bio-Rad Laboratories, Sydney, NSW, Australia). Leukocyte count was determined by a cell counter (Sysmex XT-1800i, Mundelein, IL, USA). Moreover, concentrations of CRP were determined using a solid phase, chemiluminescent immunometric assay, and concentrations of IL-6, IL-1ra, IL-1β, MCP-1, and were analyzed with a sandwich enzyme immunoassay technique, according to manufacturer’s instructions (ELISAkit, Melbourne, VIC, Australia and Merk Millipore, Billerica, MA, USA). The lower limit of quantification for the IL-6, IL-1ra, IL-1β, and MCP-1 assays were 0.9, 8.3, 0.8, and <10 pg/mL, respectively.

### Statistical Analysis

All data are reported as mean ± SD. Normal distribution was determined by Shapiro–Wilk’s test, and non-normally distributed data (inflammatory data) were logarithmically transformed prior to analysis. Repeated measures analysis of variance (ANOVA) was used to determine within- and between-group differences (YSM vs. YNS, MSM vs. MNS, smokers and non-smokers). Where a group interaction resulted, simple main effects test were applied to determine the source of significance. Significance was set at *P* < 0.05. All statistical procedures were performed using Predictive Analytic Software (PASW) (Statistical Package for the Social Sciences for Windows version 18.0, Chicago, IL, USA). An *a priori* power analysis was completed using G*Power (G*Power for Windows, version 3) based on data obtained from previous similar studies. The output parameters demonstrate a sample size of 16 to provide actual power of 0.67, and as such we recognize the potential limitation of reduced power of this study.

## Results

Baseline variables for body composition, blood lipid profile, anthropometric, and smoking variables are reported in Table 1. The MSM had significantly greater smoking history in terms of years of smoking and pack years (*P* < 0.05), but did not differ in terms of dependence level (FTND). The middle-aged cohort (MSM, MNS) demonstrated higher absolute (SM) and relative fat mass (SM and NS) than their younger counterparts (*P* < 0.05). As expected, there were significant differences for age between the middle-aged and young groups (*P* < 0.05), although no differences were evident for VO_2peak_ (*P* > 0.05). For the 40-min cycle ergometry bout, exercise-induced HR responses (% of HR_max_) were not significantly different between groups (74.2 ± 9.7, 75.6 ± 4.9, 79.6 ± 5.1, and 78.6 ± 7.2% for YSM, MSM, YNS, and MNS, respectively, *P* > 0.05). Furthermore, there were no significant differences in session RPE between groups (5.3 ± 1.6, 4.5 ± 1.8, 4.8 ± 1.5, and 4.5 ± 1.4% for YSM, MSM, YNS, and MNS, respectively, *P* > 0.05).

### Young Smokers and Non-Smokers

**Figure 1:**
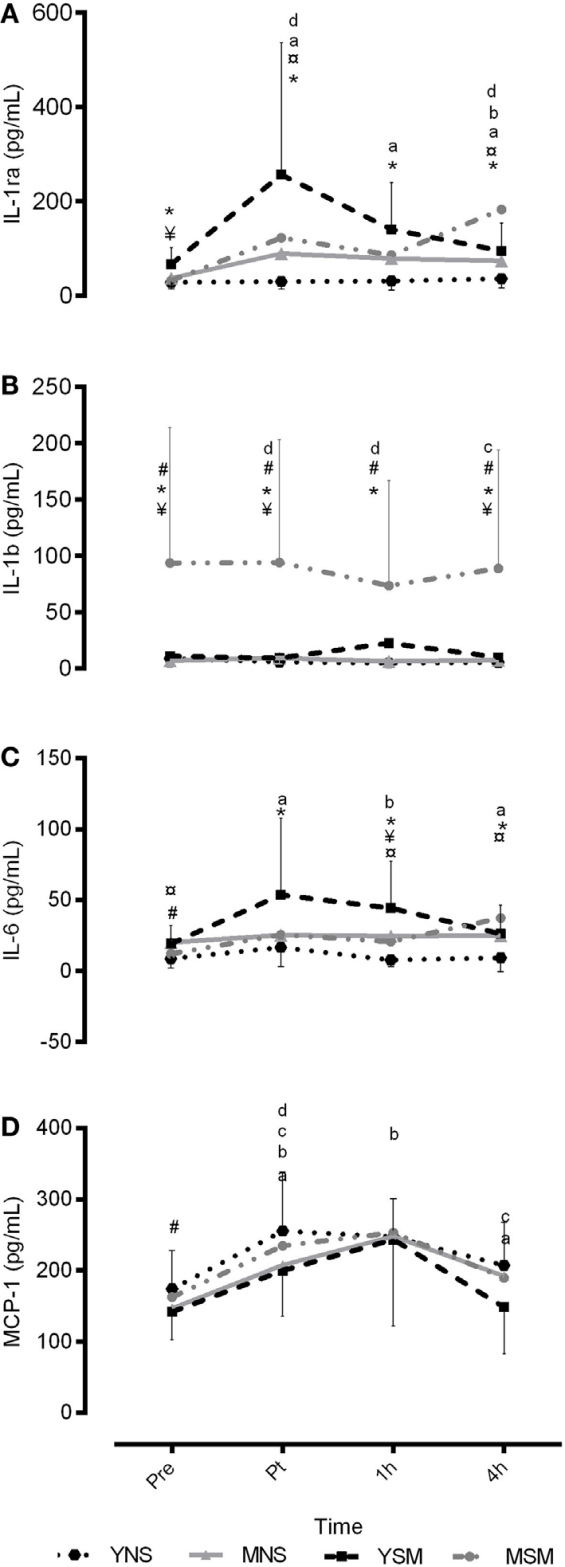
**Mean ± SD inflammatory responses for IL-6, IL-1ra, IL-1β, and MCP-1 pre, post, 1 and 4 h post exercise for younger and middle-aged smokers and non-smokers**. *represents significantly different between YSM and YNS (*P* < 0.05), ^#^ represents significantly between MSM and MNS (*P* < 0.05), ^¥^ represents significantly different between YSM and MSM (*P* < 0.05). ^¤^ represents significantly different between YNS and MNS (*P* < 0.05). Within changes for YSM, YNS, MSM, and MNS are represented by **(A–D)**, respectively (*P* < 0.05).

Inflammatory responses of IL-6, IL-1β, MCP-1, and IL-1ra are presented in Figure 1. YSM showed higher IL-1ra than YNS at baseline (*P* < 0.05), though no further baseline differences were evident. In response to an acute bout of exercise, YSM showed an amplified IL-6 response from immediately- to 1 h post compared to YNS (*P* < 0.05), alongside significantly higher IL-1ra values across all time points (*P* < 0.05). Furthermore, IL-1β was elevated at 1 h post-exercise in YSM (*P* < 0.05), which was not observed in YNS (*P* > 0.05). Moreover, the YSM group observed within-condition increases in IL-6, MCP-1, and IL-ra post-exercise (*P* < 0.05), and although IL-1ra values decreased from 1 to 4 h, they remained above pre-values (*P* < 0.05). Similarly, values for MCP-1 declined in YSM, as well as the YNS, from 1 to 4 h (*P* < 0.05). YNS observed a decline in IL-6 values from post to 1 h post (*P* < 0.05), although no significant within change was observed for IL-1ra in YNS, values were elevated above that pre-values at 4 h post-exercise (*P* < 0.05).

**Figure 2:**
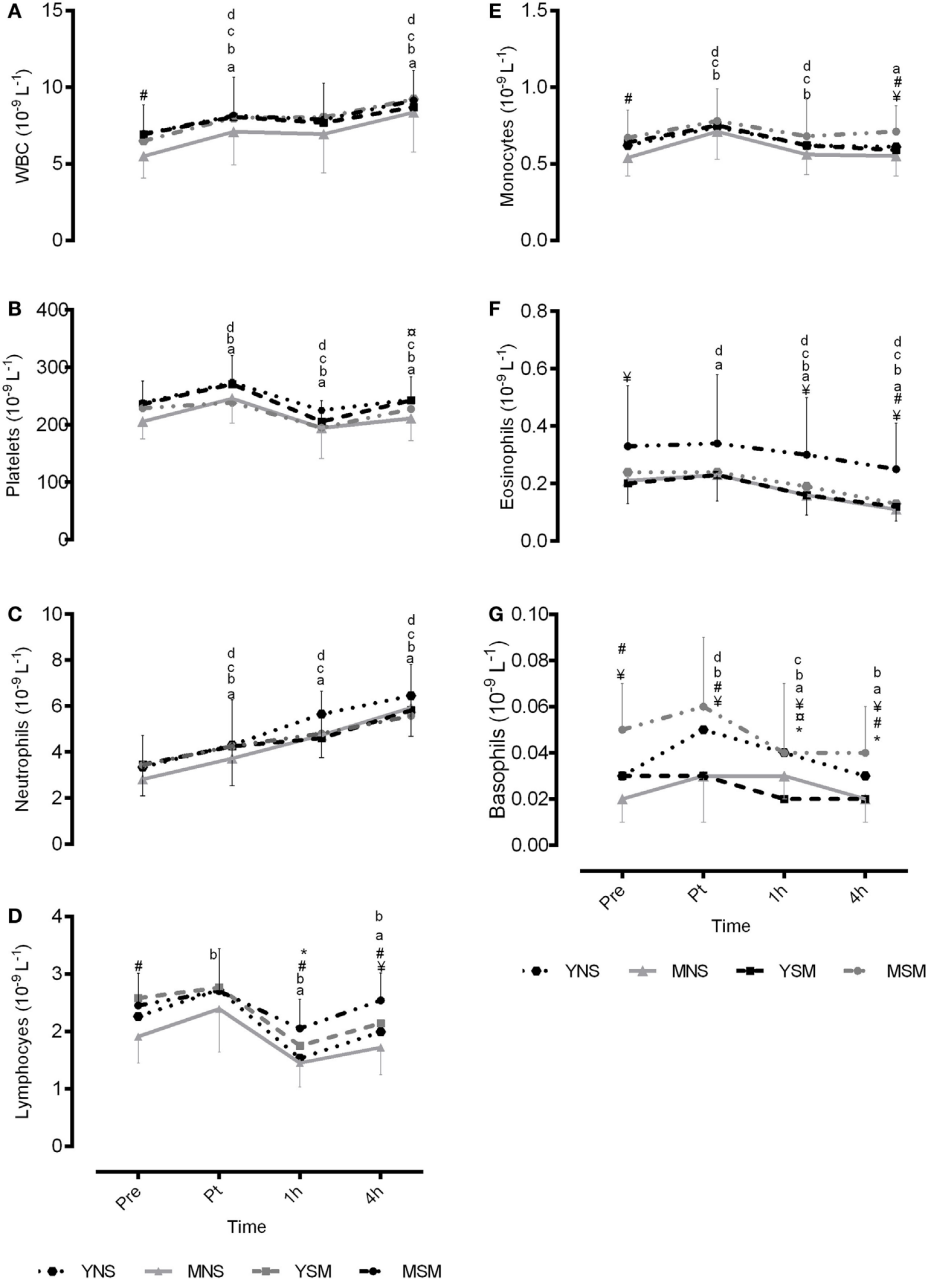
**Mean ± SD total and sub-population leukocyte count pre, post, 1- and 4-h post exercise for younger and middle-aged smokers and non-smokers**. *represents significantly different between YSM and YNS (*P* < 0.05), ^#^ represents significantly between MSM and MNS (*P* < 0.05), and ^¥^ represents significantly different between YSM and MSM (*P* < 0.05). ^¤^ represents significantly different between YNS and MNS (*P* < 0.05). Within changes for YSM, YNS, MSM, and MNS are represented by **(A–G)**, respectively (*P* < 0.05).

There were no differences between YNS and YSM at baseline or across the protocol for total leukocyte count, neutrophils, platelets, monocytes, or eosinophils (*P* > 0.05; Figure 2). YSM had higher concentrations of lymphocytes at 1 h post-exercise compared to YNS (*P* < 0.05), although basophils were higher at 1 and 4 h post-exercise in YNS compared to YSM (*P* < 0.05). Both YSM and YNS observed respective within-group increases in total leukocytes from pre- to post-exercise, a prolonged post-exercise elevation (1–4 h) in YNS (*P* < 0.05). Similarly, both YSM and YNS observed post-exercise elevation in platelets (*P* < 0.05), followed by a decline at 1 h (*P* < 0.05) and an increase thereafter (*P* < 0.05). All groups observed post-exercise elevations in neutrophil concentrations, which remained elevated at 4 h-post exercise (*P* < 0.05). YNS showed increased post-exercise lymphocyte concentrations that were not observed in the YSM cohort. Monocytes for YNS increased post-exercise followed by a decline thereafter, while YSM values were below pre-values at 4 h post-exercise (*P* < 0.05). Basophils and eosinophils for YSM and YNS both resulted in declines from post-exercise (*P* < 0.05).

### Middle-Aged Smokers and Non-Smokers

At baseline, MSM had lower IL-6 when compared to MNS (*P* < 0.05). Exercise induced no within-group changes for IL-6 in either MSM or MNS. Both groups observed post-exercise increases in IL-1β followed by a decline to 1 h (*P* < 0.05); however, MSM experienced marginal elevations in post-exercise IL-1β (Post-4 h) (*P* < 0.05). Furthermore, both MNS and MSM observed post-exercise elevations in MCP-1 (*P* < 0.05), which remained elevated to 1 h for MNS (*P* < 0.05), though declined for MSM (*P* < 0.05). Finally, MNS observed an increase in IL-1ra post exercise, which remain elevate above pre at 4 h post-exercise (*P* < 0.05) that was not observed in MSM (*P* > 0.05).

There were no baseline differences for neutrophils or platelets in the middle-aged cohort (*P* > 0.05). Total leukocyte count was higher at baseline for MSM compared to MNS (*P* > 0.05). Furthermore, MSM demonstrated higher lymphocyte concentrations at baseline and 1 and 4 h post-exercise (*P* > 0.05). In addition, MSM had higher concentrations of monocytes and basophils at baseline and 4 h post-exercise (*P* > 0.05) compared to MNS. Moreover, eosinophils for MSM were elevated above MNS at 4 h post-exercise (*P* > 0.05). Both MSM and MNS observed a within-group increase in total leukocytes from pre- to post-exercise (*P* < 0.05), which was further increased from 1 to 4 h post-exercise (*P* < 0.05). Similarly, MNS observed post-exercise elevation in platelets (*P* < 0.05) followed by a decline at 1 h (*P* < 0.05), which was not observed in MSM (*P* > 0.05). Both MNS and MSM showed post-exercise elevations in neutrophil concentrations, which remained elevated 4 h-post exercise (*P* < 0.05). MNS showed increases in lymphocyte concentrations post-exercise, not observed in the MSM cohort, who experienced a decline post-exercise (post-1 h) followed by an increase at 4 h (*P* < 0.05). Monocytes for MNS and MSM increased post-exercise followed by a decline thereafter (*P* < 0.05). Basophils for MNS increased post-exercise, yet MSM decline from post to 1 h (*P* < 0.05). Finally, eosinophils for MNS increased post-exercise, however, declines were observed post exercise for MSM (post–1 h) (*P* < 0.05).

### Smokers

For the smoker cohort, MSM had higher IL-1β at baseline when compared to YSM; moreover, IL-1ra values were greater for YSM at baseline (*P* < 0.05); yet, IL-1β and MCP-1 were higher at 4 h post-exercise for MSM than YSM (*P* < 0.05). There were no between-group differences for YSM and MSM for total leukocyte count, platelet, or neutrophils (*P* > 0.05). However, MSM demonstrated higher lymphocytes and monocytes at 4 h post-exercise (*P* < 0.05). Furthermore, eosinophils were higher in MSM at baseline and 1 and 4 h post-exercise (*P* < 0.05) and basophils were higher in MSM across all time points (*P* < 0.05).

### Non-Smokers

MNS had higher IL-6 at baseline than YNS, while no baseline differences were evident MCP-1, CRP, IL-1β, or IL-1ra for the two non-smoking groups (*P* < 0.05). Exercise-induced elevations in IL-6 in MNS (1 and 4 h) were greater than YNS (*P* < 0.05). Furthermore, a greater post-exercise (4 h post) increase in IL-1ra was evident in MNS compared to YNS (*P* < 0.05). There were no between-group differences for total leukocyte count neutrophils, lymphocytes, monocytes, or eosinophils between MNS and YNS (*P* > 0.05). Platelets at 4 h were higher in YNS, as were basophils at 1 h post-exercise (*P* > 0.05).

## Discussion

This study demonstrated the effects of acute exercise on markers of immune and inflammatory function in smokers and non-smokers and between longer (MSM) and shorter (YSM) smoking history. Accordingly, middle-aged smoker’s exhibit elevated basal concentrations of MCP-1 than their age-matched non-smoker counterparts and a younger smoker group, which increases with smoking history and may indicate smoking associated endothelial injury and future systemic disease risk. Furthermore, a novel finding of this study is the marked differences between smokers and their non-smoker counterparts; specifically, while exercise had a stimulatory effect on markers of inflammation for YSM and MSM, an amplified response was evident in YSM. Contrastingly, the post-exercise leukocyte response was greater in MSM compared to YSM and non-smokers, suggestive of immune and inflammatory changes reflective of smoking history.

Tobacco smokers are predisposed to an array of non-communicable diseases, with this susceptibility thought to arise through alterations in immune and inflammatory process from the repeated exposure to tobacco smoke (22–24). As evidence, immunosuppression and a heightened inflammatory profile consistent with smoking history are common features observed in chronic tobacco smokers (4, 6). However, the complexity of the composition of tobacco smoke in addition to factors, such as smoking duration, frequency, gender, and environmental factors, poses difficulty when interpreting immune-inflammatory system responses (2, 25–27). As evidence, Kuschner et al. (5) revealed that concentrations of MCP-1, IL-1β, IL-6, TNF-α, and IL-8 were elevated in the bronchoalveolar lavage (BAL) of smokers when compared to non-smokers. Findings from the present study demonstrate that smokers have elevated basal concentrations of the chemokine MCP-1 in peripheral blood, which is linked to inflammatory cell recruitment and has been previously demonstrated in the pulmonary microenvironment (28). However, elevations in MCP-1 in chronic smokers as observed in the present study may be associated with the inflammatory load linked to long-term smoking and could reinforce the relationship between smoking and immune and inflammatory alterations.

In addition to elevations in inflammatory cytokines, elevated concentrations of circulating leukocytes have been implicated as a predictive marker of lifestyle-related disease (29, 30). Smoking is reported to elevated leukocyte count, with Frohlich et al. (4) reporting white blood cell (WBC) count to be positively associated with smoking status in men. Furthermore, Kawada (31) reported current smokers to have elevated WBC count above that of non-smokers. The present study also demonstrated higher baseline WBC in the MSM group, along with elevated basal concentrations of lymphocytes and monocytes. In agreement with previous literature (4, 31), the current findings suggest that prolonged exposure to smoking insult can induce elevated total and fractional leukocyte counts, and escalate the inflammatory risk.

While the effects of long-term smoking are well established, less well known are the effects of exercise in a smoker population, particularly given the supposed important role exercise has as an anti-inflammatory mediator. A novel finding from this study is that YSM demonstrated a greater exercise-induced inflammatory response than YNS, as evidenced by the observed elevations in IL-6 and IL-1ra. Interestingly, YSM also exhibited a greater peak in IL-1β at 1 h post compared to YNS. To explain these counter-intuitive observations, post-exercise declines in IL-1ra and increased IL-1β at 1 h may be a result of receptor availability, as when concentrations of IL-1ra decline, IL-1β is able to bind with the IL-1 receptor and exert subsequent biological actions (32). Regardless, the current study indicates that a moderate-intensity exercise stimulus was not sufficient to induce a significant inflammatory response in healthy non-smokers; hence, future studies should consider a higher intensity to induce a greater cytokine response in non-smokers. Furthermore, chronic exposure to tobacco smoke may prime immune and inflammatory cells in YSM (33), thereby inducing a greater inflammatory response to a similar exercise stimulus. However, an observed immunosuppression seems evident in MSM, and thus an inhibition of the exercise-induced inflammatory response was evident (14).

An acute bout of exercise is suggested to elicit an influx of cytokines with anti-inflammatory actions (12); interestingly, the magnitude of response in MSM was lower than YSM for the same exercise stimulus. More specifically, MSM observed elevated IL-1β without concurrent elevations on IL-1ra or IL-6, which is suggestive of a delayed or compromised immune-inflammatory response to exercise. The present study suggests that MSM have elevated IL-1β, and furthermore, that the typical inflammatory response of elevated IL-1ra and IL-6 (12) to exercise was not observed. It could be hypothesized that such changes to the inflammatory profile and response in MSM may be attributed to changes in the T-helper (Th)-1 and T-helper (Th)-2 cytokine balance. The Th-1/Th-2 cytokine balance theory implicates Th-1- and Th-2-derived cytokines in the regulation of homeostatsis; chronic tobacco smoke exposure is suggested to cause a shift in the cytokine balance, and thus immune regulation (34, 35). Moreover, long-term smoking is suggested to induce alterations to the responsiveness of the hypothalamic–pituitary–adrenal (HPA) axis (14, 36). The containment of inflammation is partly mediated by the HPA axis; consequently, a smoking-induced decrease in the responsiveness of the HPA axis may promote the disinhibition of immune and inflammatory reactions (36). Although these mechanisms were not measured in the current study, they may provide explanation for the compromised anti-inflammatory response to exercise observed in MSM.

Regulatory immune and inflammatory mediators are known to increases in response to an exercise stimulus (7, 37), particularly, circulating leukocytes neutrophils, lymphocytes, and monocytes are reported in increase in a biphasic fashion (7, 37). In the present study, MSM demonstrated greater concentrations of lymphocytes, basophils, and monocytes post-exercise than young smokers and non-smokers. By contrast, Ceddia et al. (38) reported attenuated leukocytosis in response to maximal exercise in older vs. younger participants – although no literature exists on the effects of smoking on such responses. Moreover, YSM demonstrated higher lymphocytes at 1 h post-exercise but lower basophils until 4 h post-exercise compared with YNS. Circulating leukocytes, particularly monocytes are thought to play a central role in inducing the acute phase response (37) and are the source of the cytokines TNF-α, IL-6, and IL-1. Therefore, it is plausible to suggest that changes in circulating leukocytes may be responsible for the altered inflammatory response to exercise in a smoker population. However, further studies are needed to confirm such a hypothesis.

In conclusion, this present study revealed that smoker’s exhibit elevated MCP-1 and IL-1β that seem to be increased with a longer smoking history (~15 years). Although such findings may be attributed to age, the lack of significant difference among NS indicates that possibly such differences between smokers may be due to length of smoking history. In response to an acute bout of exercise, the SM and NS presented marked differences; specifically, tobacco smoke may prime circulating leukocytes in YSM, resulting in an amplified inflammatory response to the exercise stimulus when compared to their NS counterparts. Moreover, the immune-inflammatory responses of MSM may indicate an effect of chronic exposure, via elevated WBC count and a compromised inflammatory response to exercise.

## Author Contributions

TK, RD, and FM were involved in the methodological design. TK collected and analyzed the data and drafted the manuscript. RD and FM provided critical feedback on the manuscript.

## Conflict of Interest Statement

The authors declare that the research was ­conducted in the absence of any commercial or financial relationships that could be construed as a potential conflict of interest. The Review Editor, Daisuke Kamimura, and the Handling Editor, Masaaki Murakami, declare their shared affiliation and recent collaborations and the Handling Editor states that the review process nevertheless met the standards of a fair and objective review.
